# zk-Guard-R: Policy-Hidden and Replay-Safe zk-SNARK Access Control for IoT Sensor Data Stored on IPFS

**DOI:** 10.3390/s26165045

**Published:** 2026-08-08

**Authors:** Huiying Hou, Yucong Ma, Zisu Zhao, Xuerui Gan

**Affiliations:** 1 The Key Laboratory of Grain Information Processing and Control, Henan University of Technology, Zhengzhou 450001, China; 2 The School of Artificial Intelligence and Big Data, Henan University of Technology, Zhengzhou 450001, China; 3 The State Key Laboratory of Mathematical Engineering and Advanced Computing, Henan Province Key Laboratory of Cyberspace Situation Awareness, Zhengzhou Institute of Information Science and Technology, Zhengzhou 450001, China; 4 The School of Electrical Engineering, Henan University of Technology, Zhengzhou 450001, China

**Keywords:** Internet of Things, sensor data sharing, zero-knowledge proofs, zk-SNARKs, access control, blockchain, IPFS, edge gateway, vector commitments, range proofs

## Abstract

IoT sensor deployments increasingly export measurement streams to edge gateways and content-addressed storage such as IPFS, but access control decisions must be enforced without disclosing sensor owner policies, requester attributes, or stale data versions. Existing blockchain, CP-ABE, and zero-knowledge approaches reduce parts of this leakage, yet they can still expose public policy structure, accept stale Merkle proofs after sensor stream updates, overload provers when policies grow, or leave IPFS gateways vulnerable to bandwidth abuse. This paper proposes zk-Guard-R, a policy-hidden and replay-safe zk-SNARK access control framework for privacy-preserving IoT sensor data sharing. zk-Guard-R replaces public sparse policy matrices with MiMC-Merkle policy commitments verified inside the proof, separates long-lived logical sensor policy roots from frequently updated physical IPFS data roots, binds every proof to an on-chain nonce, and decouples attribute possession from policy interpretation through a bounded stack-based policy interpreter. Numeric sensor-access predicates are represented through committed values and range check gadgets, while an off-chain verification gateway couples accepted proofs with payment channel vouchers before releasing encrypted IPFS chunks. The design contribution is separated from the measured prototype: the full protocol specifies a bounded policy interpreter, whereas the present gnark prototype evaluates the core committed policy, committed attribute, range check, data root, nonce, Solidity verifier, and gateway-metering mechanisms. We implement a gnark BN254/Groth16 research prototype and benchmark it against a matrix-public zk-Guard prototype, a blockchain ABAC baseline, an IoT token/HMAC baseline, and a CP-ABE-style cryptographic-work proxy. For 128 attributes, the zk-Guard-R prototype with MiMC-Merkle commitments uses 425,574 R1CS constraints, generates proofs in 3.12 s, verifies in 0.73 ms, and uses 641 MB peak Go heap allocation. A three-run repeat of the 128-attribute configuration gives a proof-generation mean of 2.80 s with a 0.54 s standard deviation on the same local host, illustrating the runtime variability of prover measurements. We also deploy the generated Solidity verifier on a local Anvil EVM and measure 241,942 gas for a successful verification transaction, and we evaluate a local Kubo/IPFS gateway under valid, replayed, and voucher-limited flood requests. The results show that zk-Guard-R shifts substantial but measurable work to the prover while improving policy confidentiality, freshness, and gateway metering for IPFS-backed IoT sensor data sharing.

## 1. Introduction

IoT deployments increasingly collect environmental, industrial, health, mobility, and smart-city measurements through heterogeneous sensors and edge gateways. These data streams are valuable precisely because they can be shared across organizations, but they are also sensitive: a sensor reading can reveal location, equipment state, user behavior, or operational schedules. IPFS is attractive in this setting because sensor records can be chunked, content-addressed, cached near edge consumers, and retrieved without a single storage operator [1,2]. However, content addressing alone does not solve authorization. A sensor owner can encrypt chunks or register policy decisions on a blockchain, but the policy-checking process itself should not reveal requester attributes, facility roles, sensor-group topology, or internal authorization rules.

Blockchain-based access control addresses auditability by executing authorization logic in smart contracts, but public ledgers make both attributes and policies observable. Ciphertext-policy attribute-based encryption (CP-ABE) supports fine-grained authorization [3,4], while recent blockchain-assisted designs improve update and audit workflows. However, CP-ABE often couples the access policy to ciphertext and decryption cost, making frequent sensor stream updates and large attribute universes expensive. Zero-knowledge access control offers a different route: the requester proves that private credentials satisfy a sensor owner policy, and the chain or edge gateway verifies only a succinct proof. The original zk-Guard framework follows this route by combining Groth16 zk-SNARKs [5] with a universal policy circuit, sparse policy configuration matrices, and Merkle tree proofs (MTPs) for IPFS chunks [6]. Its central insight is valuable for IoT: the circuit should be reusable while sensor owners update policies and content roots.

The same design, however, leaves several production-level security gaps. First, a sparse configuration matrix can be semantically label-hidden but still structure-revealing: an observer can infer policy size, sparsity, role hierarchy, and policy-update patterns. Second, the MTP that links a requested IPFS chunk to a root block is not by itself a freshness guarantee. If a content identifier changes after an update, a stale proof tied to an old root can remain meaningful unless the authorization proof is bound to a current on-chain version. Third, compiling rich policies into tree-shaped constraints can push complexity to the prover when policies include nested Boolean expressions, range predicates, or frequent updates. Fourth, a gateway that releases IPFS chunks after proof verification can still be flooded by high-volume requests unless each accepted request carries an economic cost.

This paper proposes zk-Guard-R, a resilient redesign of zk-SNARK access control for IoT sensor data stored on IPFS. The design keeps the original goal of decoupling circuits from policies, but changes what is public, what is versioned, and what the prover executes. Instead of publishing a sparse policy matrix, zk-Guard-R stores only a policy commitment. Instead of using a single content root, it separates a long-lived logical sensor policy root from a frequently updated physical IPFS data root. Instead of compiling each policy into a tree-shaped circuit, it verifies attributes in one shallow module and evaluates a private reverse-Polish policy bytecode in a bounded stack interpreter. Finally, instead of treating bandwidth as a free post-verification resource, it places a payment channel voucher in front of gateway release.

To isolate the novelty more precisely, we distinguish between three roles of the paper. The original mechanisms are the combination of hidden policy commitments with nonce-bound double roots for IPFS-backed sensor streams, the use of a bounded private policy interpreter as the target access relation, and the coupling of accepted gateway release to voucher state. The adapted mechanisms are standard Merkle commitments, Groth16 proofs, range check gadgets, and payment channel accounting. The engineering integration is the gnark, Solidity, and Kubo/IPFS prototype that tests the main commitment, freshness, range, verifier gas, and gateway-metering surfaces under one reproducible workflow.

The contributions are as follows:

1. We identify a policy-side-channel problem in public sparse policy encodings and introduce a policy commitment layer in which the policy matrix or bytecode is a private witness while only a commitment is stored on-chain.
2. We define a double root freshness model that binds proofs to a logical policy root, a current IPFS data root, and a registry nonce, making stale MTPs unverifiable immediately after an authorized root update.
3. We design a two-plane proving architecture: an attribute plane proves possession and range predicates over committed attributes, while a bounded stack interpreter evaluates private policy bytecode without regenerating setup material for each policy shape.
4. We integrate a verification gateway with payment channel vouchers, converting bandwidth exhaustion from a free attack into a costed interaction while preserving off-chain proof verification for edge gateways.
5. We implement a reproducible gnark BN254/Groth16 prototype for the IoT access statement and report constraint counts, proof time, verification time, memory use, deployed Solidity verifier gas, Kubo/IPFS gateway stress results, and comparisons against zk-Guard-style, CP-ABE-style, blockchain ABAC, and IoT token baselines.

The contribution claims are therefore calibrated as follows. The implemented prototype supports the committed-vector relation, range predicate, data root freshness, nonce binding, Solidity verification, and gateway stress tests. The bounded reverse-Polish policy interpreter is specified at the protocol and cost-model level, but its full instruction-decoding and stack-transition circuit is not included in the current benchmark tables; implementing and benchmarking this full interpreter is identified as a remaining engineering task.

The paper is organized as follows. Section 2 reviews the relevant literature. Section 3 gives the system and threat model. Section 4 presents the zk-Guard-R protocol. Section 5 analyzes security properties. Section 6 gives complexity and prototype evaluation, and Section 7 concludes.

## 2. Related Work

### 2.1. Blockchain-Based Access Control for IoT and Decentralized Storage

Smart contracts provide a tamper-evident execution layer for authorization decisions, and they have been widely used in IoT, cloud, and decentralized storage scenarios [7,8,9]. In IPFS-based IoT designs, the blockchain typically records access control metadata while IPFS stores encrypted sensor chunks or short time-window aggregates. This separation avoids storing high-volume measurements on-chain, but the transparent ledger can reveal requester attributes, policy structures, and update patterns. Moreover, if every sensor chunk triggers a chain transaction, the storage protocol loses the concurrency advantage that made IPFS attractive for edge data distribution.

zk-Guard addresses this by verifying a zk-SNARK proof once and using MTPs to connect sub-blocks to an authorized root [6]. The mechanism reduces repeated policy checks, but its public sparse policy configuration remains observable. zk-Guard-R therefore focuses on hiding the policy representation itself and on binding chunk proofs to a current data version.

### 2.2. Attribute-Based Encryption and Hidden Access Policies

CP-ABE allows a ciphertext to encode a policy and a user’s secret key to encode attributes [3,4]. Later work introduced partially hidden access structures and more efficient constructions [10,11]. These schemes are useful when decryption should enforce authorization without an online verifier. In dynamic decentralized storage, however, CP-ABE may require expensive ciphertext or key updates when policies or attributes change. Blockchain-assisted CP-ABE improves auditability, but it still faces the problem that policy and ciphertext are tightly coupled.

zk-Guard-R uses a different design point. Access is proved by a zero-knowledge statement over committed requester attributes and a committed sensor owner policy, while the IPFS data root can be updated independently as a sensor stream evolves. This is better suited to settings where policy evaluation should be private, publicly verifiable, and frequently refreshed.

### 2.3. Succinct Zero-Knowledge Proofs and Program Interpreters

zk-SNARKs give short proofs and efficient verification for arithmetic circuit satisfiability. The SNARKs-for-C line showed how machine executions can be proved succinctly by compiling bounded program executions into constraints [12]. Groth16 further reduced proof size and verifier work for pairing-based non-interactive arguments [5]. These systems are attractive for blockchain verification because public-chain verifiers can check a fixed number of group and pairing equations.

The cost is that the circuit must encode the computation. If every policy shape is compiled directly, policy evolution can require new setup material or larger circuits. Universal circuits avoid policy-specific setup, but public configuration may leak structure. zk-Guard-R adopts a bounded interpreter: the circuit verifies execution of a small policy virtual machine for up to $L_{\max}$ instructions. The circuit is constant with respect to a particular policy shape, but not with respect to the configured maximum instruction budget.

### 2.4. Commitments, Range Proofs, and Payment Channels

Commitment schemes allow a party to bind to data while hiding it. KZG polynomial commitments provide constant-size polynomial commitments and openings under pairing assumptions [13]; Merkle commitments provide transparent hash-based commitments with logarithmic openings [14]. For in-circuit verification, hash-based commitments built from SNARK-friendly permutations are often preferable to KZG openings because pairing verification inside an arithmetic circuit is expensive.

Numeric predicates require more than equality to a hash. Pedersen commitments are additively homomorphic and information-theoretically hide under standard assumptions [15]. Bulletproofs popularized efficient range proofs for committed values without a trusted setup [16]. In a Groth16 circuit, the same design idea can be represented as bit decomposition and range check gadgets over committed attributes.

Finally, payment channels move repeated payments off-chain and settle only disputes or final balances on-chain. Lightning-style channels [17] and anonymous payment channels [18] show that tiny per-request payments can be enforced with low ledger overhead. zk-Guard-R uses this idea as a bandwidth-abuse defense at the data gateway.

## 3. System and Threat Model

### 3.1. Entities

zk-Guard-R contains seven entities. The *attribute issuer* authenticates requesters and issues signed attribute commitments, for example organization, device class, duty role, or consent status. The *sensor owner* uploads encrypted sensor chunks or time-window aggregates to IPFS, defines access policies, and registers commitments and roots. The *data requester* may be a mobile client, edge analytics service, or emergency-response application that holds private attributes and generates access proofs. The *on-chain registry* stores only commitments, verification keys, roots, and nonces. The *prover* is the requester’s local proof-generation component. The *edge verification gateway* checks proofs and payment vouchers before releasing chunks. The *IPFS provider* stores and serves content-addressed sensor chunks.

### 3.2. Adversary

We consider a probabilistic polynomial-time adversary that can observe the blockchain, read all public registry state, submit malformed access requests, replay old proofs, inspect policy-update timing, and operate malicious IPFS nodes or gateway clients. The adversary may control some requesters and may obtain old sensor content identifiers. The adversary cannot break the binding and hiding properties of the chosen commitment scheme, forge issuer signatures, find hash collisions, or produce false accepting zk-SNARK proofs except with negligible probability.

We additionally make the trust and leakage boundaries explicit. A compromised issuer can issue false attributes and is outside the protection of the proof relation unless issuer keys are revoked in the registry. Colluding requesters can pool their legitimately issued attributes only if the issuer credential format permits delegation; the intended instantiation binds attributes to requester keys. Observers may learn metadata such as policy-version timing, accessed sensor identifiers, proof submissions, voucher activity, configured public bounds, and gateway availability events. These metadata are not hidden by zk-Guard-R and are included in the leakage function in Section 5.

The gateway is assumed to be economically rational and auditable. It may refuse service or attempt to overcharge, but payment channel state and signed receipts allow disputes. A malicious gateway can deny availability, so zk-Guard-R supports multiple gateways; it does not claim that one gateway alone provides censorship resistance.

For front-running and state-desynchronization, the verifier accepts a request only against the latest registry tuple returned by the contract or by a signed registry snapshot whose block height is within the configured freshness window. Voucher replay is prevented by monotonically increasing channel sequence numbers and gateway-side receipt logs. Proof verification flooding remains a network-layer and verifier-capacity issue; the voucher mechanism meters the expensive chunk release path rather than claiming to eliminate all packet-level denial of service.

### 3.3. Security Goals and Non-Goals

zk-Guard-R targets five goals:

1. **Access-decision soundness**: A proof is accepted only if the user owns valid attributes satisfying the committed policy for the current registry state.
2. **Policy confidentiality**: Public observers learn the existence and version of a policy but not the policy matrix, bytecode, sparsity pattern, or attribute labels beyond what is intentionally disclosed.
3. **Replay resistance**: Proofs and MTPs tied to old data roots or old nonces are rejected after a registry update.
4. **Attribute privacy with numeric predicates**: Equality and range predicates are proved without revealing raw attribute values.
5. **Economic DoS resistance**: Accepted chunk release consumes a signed voucher or channel balance, making large-scale bandwidth abuse costly.

The design does not guarantee deletion of sensor chunks already pinned by third parties in IPFS. Freshness prevents current authorized gateways from releasing stale versions, but cryptographic confidentiality for old pinned blocks requires encrypted chunks and versioned key rotation.

## 4. The zk-Guard-R Design

### 4.1. Design Overview

**Figure 1:**
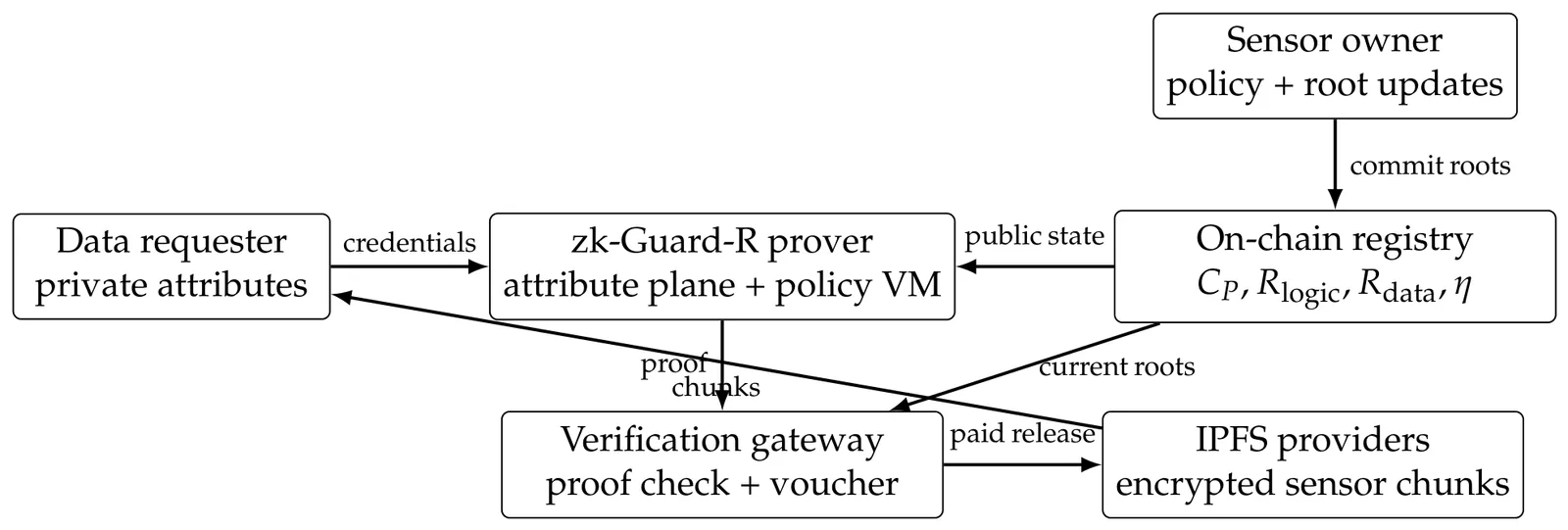
Logical architecture of zk-Guard-R for IoT sensor data sharing. The chain stores commitments and freshness state rather than public policy matrices. The policy and requester attributes are private witnesses to a zero-knowledge proof, while the edge gateway releases encrypted IPFS sensor chunks only after proof and voucher validation.

zk-Guard-R reorganizes zk-Guard into four layers (Figure 1). The registry layer stores commitments and freshness state. The proof layer checks attributes, policy bytecode, range predicates, and root freshness inside a zk-SNARK. The gateway layer verifies proofs and payment vouchers off-chain. The storage layer only releases chunks that match the current data root.

### 4.2. Notation and Registry State

Let *U* be the requester attribute universe, *P* a sensor access policy, and $B=(b_{1},\ldots ,b_{s})$ the IPFS chunk set of a sensor stream or time-window aggregate. The registry stores

$$\mathrm{Registry}\left[pid\right]=\left(C_{P},R_{\mathrm{logic}},R_{\mathrm{data}},\eta ,vk,\sigma_{SO}\right),$$

where $C_{P}=\mathrm{Commit}\left(P;\rho_{P}\right)$ is a hiding and binding commitment to the policy representation, $R_{\mathrm{logic}}$ identifies the long-lived sensor authorization object, $R_{\mathrm{data}}$ is the current Merkle root over encrypted IPFS chunk identifiers, η is a monotonically increasing version, vk is the verification key, and $\sigma_{SO}$ is the sensor owner’s signature over the tuple.

### 4.3. Policy Commitment Layer

The original sparse matrix $M_{\mathrm{conf}}$ is replaced by a private policy witness. The sensor owner computes a commitment

$$C_{P}=\mathrm{Commit}\left(\mathrm{encode}\left(P\right);\rho_{P}\right),$$

and publishes only $C_{P}$. The prover supplies encode(P) and $\rho_{P}$ privately. The circuit enforces

$$\mathrm{Commit}\left(\mathrm{encode}\left(P\right);\rho_{P}\right)=C_{P}$$

before evaluating the policy. For a Merkle commitment, the circuit either recomputes the root over a bounded policy array or verifies authenticated openings for the bytecode cells used by the interpreter. For a KZG commitment, openings are succinct outside the circuit, but in-circuit verification is costly unless the proof system natively supports the same pairing arithmetic. Therefore, the default zk-Guard-R instantiation uses a SNARK-friendly Merkle commitment, and treats KZG as a public-verifier optimization for future pairing-friendly implementations.

The confidentiality theorem below is stated for the full-root mode in which the policy program is padded to the public bound $L_{\max}$ and the circuit recomputes or verifies a fixed-shape commitment. In an optimized authenticated-opening mode, the leakage function must additionally include the number of opened cells *q*, and, if openings are not padded or accessed in a fixed schedule, the access pattern of opened bytecode locations. We therefore treat the opening mode as a performance optimization that requires padding to recover the same structure-hiding claim.

### 4.4. Double Root Freshness and Nonce Binding

zk-Guard-R separates policy validity from content freshness. $R_{\mathrm{logic}}$ changes only when the logical access object changes, for example, when a sensor owner replaces the policy family. $R_{\mathrm{data}}$ changes whenever the encrypted IPFS sensor chunk set changes. The nonce η increases on every authorized update. The public inputs to the access proof include

$$x_{\mathrm{pub}}=\left(C_{P},R_{\mathrm{logic}},R_{\mathrm{data}},\eta ,H_{A},pid\right),$$

where $H_{A}$ is the public commitment to the user’s attribute commitment set. The circuit checks that the MTP for the requested chunk reconstructs $R_{\mathrm{data}}$, and the contract or gateway checks that $R_{\mathrm{data}}$ and η match the current registry state.

### 4.5. Attribute Plane and Policy Interpreter

The proof is split into two planes. The attribute plane proves that the user owns committed attributes issued by an authorized issuer. For equality attributes, the leaf contains a commitment to a key-value pair. For numeric attributes, the leaf contains a Pedersen-style commitment $C_{x}=g^{x}h^{r}$, and the circuit verifies a range predicate by bit decomposition:

$$x-a=\sum_{i=0}^{k-1}2^{i}z_{i},\;z_{i}\left(1-z_{i}\right)=0,\;0\leq x-a<2^{k}.$$

This supports statements such as Dept=Finance and Age≥18 without revealing the raw age.

The policy plane executes a bounded reverse-Polish bytecode program. The instruction set is deliberately small:

{PUSH_ATTR,EQ,RANGE,AND,OR,NOT,HALT}.

The circuit contains a stack of size $S_{\max}$ and a loop of length $L_{\max}$. Each step fetches one committed instruction, decodes it by one-hot selector constraints, updates the stack, and enforces that HALT leaves a root value equal to 1. The circuit is independent of the syntactic tree of a particular policy, although the chosen $L_{\max}$ bounds the maximum policy length.

The public bounds $L_{\max}$ and $S_{\max}$ are deployment parameters rather than hidden values. They should be selected from the largest policy class supported by the deployment; larger bounds improve policy flexibility but increase prover cost even for short policies. In the current gnark prototype, we instantiate a simpler private policy vector circuit rather than the full bytecode VM. This lets us measure the dominant MiMC-Merkle, range, data root, nonce, Solidity verifier, and gateway components without claiming that the full interpreter circuit has already been benchmarked.

### 4.6. Gateway and Payment Channel Release

The gateway accepts a request only if it contains: (i) the proof π, (ii) the public state tuple $\left(pid,R_{\mathrm{logic}},R_{\mathrm{data}},\eta \right)$, (iii) an MTP for the requested chunk, and (iv) a signed payment channel voucher $v_{j}$ with sequence number *j*. The gateway first checks that the channel has sufficient locked balance and that the voucher sequence number is fresh. It then verifies the proof against the current registry state. If verification succeeds, it releases the requested encrypted chunk and records a signed receipt. The final channel settlement charges only accepted releases; invalid proofs can be rate-limited by requiring a refundable verification deposit or by charging a small verification voucher.

### 4.7. Protocol Algorithms

Table 1 summarizes the main procedures. The algorithms are written at the interface level so that different SNARK backends, commitment schemes, and channel systems can instantiate them.

## 5. Security Analysis

**Definition** **1** (Access-decision soundness)**.** *An adversary wins the access-decision soundness game if it outputs a proof π and public input $x_{\mathrm{pub}}$ accepted by Vrfy, but there is no valid issued attribute set, policy opening, range-witness assignment, and current data root proof satisfying the zk-Guard-R relation.*

**Theorem** **1.** *If the underlying zk-SNARK is knowledge-sound, the commitment scheme is binding, the issuer signature is unforgeable, and the hash function used for Merkle roots is collision-resistant, then zk-Guard-R satisfies access-decision soundness.*

**Proof.**  Assume an adversary produces an accepting proof for a false access decision, as shown in Table 2. Knowledge soundness yields a witness satisfying the arithmetic relation. The relation includes issuer-signature verification or a membership proof under an issuer-authorized attribute root; otherwise, signature unforgeability is broken. It includes the equality $C_{P}=\mathrm{Commit}\left(P;\rho_{P}\right)$; if the adversary uses a different policy from the committed one, binding is broken. It includes range and Boolean interpreter constraints; therefore, the policy result must equal 1 for the extracted attributes. It also includes reconstruction of $R_{\mathrm{data}}$ from the chunk MTP; if an invalid path is accepted, collision resistance is broken. Thus an accepting proof for a false decision contradicts at least one assumed primitive.    □

**Definition** **2** (Policy confidentiality leakage)**.** *Let L(P) denote the public leakage consisting of the policy identifier, commitment $C_{P}$, verification key identity, configured bounds $\left(L_{\max},S_{\max}\right)$, registry update times, proof submission times, requested sensor identifiers, voucher metadata, and gateway availability events. In the full-root padded mode, L excludes policy labels, Boolean structure, sparsity pattern, and the actual bytecode length below $L_{\max}$. In authenticated-opening mode, L additionally includes the number of opened cells and any unpadded opening pattern.*

**Definition** **3** (Policy confidentiality)**.** *Policy confidentiality holds if any two policies $P_{0}$ and $P_{1}$ satisfying $L\left(P_{0}\right)=L\left(P_{1}\right)$ induce computationally indistinguishable public views for any chain observer.*

**Theorem** **2.** *In the full-root padded mode, if the policy commitment is hiding and the zk-SNARK is zero-knowledge, then zk-Guard-R hides policy labels, contents, Boolean structure, sparsity pattern, and actual bytecode length up to the leakage function L.*

**Proof.**  The public registry contains $C_{P}$, roots, nonces, verification keys, and public bounds, but not *P*, its sparse matrix, or its padded bytecode. Hiding of $C_{P}$ makes commitments to $P_{0}$ and $P_{1}$ indistinguishable when their leakage functions match. The proof transcript reveals no witness information beyond the truth of the relation by zero knowledge. Since the full-root mode pads the program to $L_{\max}$ and evaluates a fixed-shape circuit with stack bound $S_{\max}$, the public circuit and transcript do not expose the actual policy tree, sparsity, labels, or sub-bound bytecode length. The remaining metadata listed in L are explicitly outside the hidden witness and must be mitigated, if necessary, by operational measures such as batching, cover traffic, and fixed-length update windows.    □

**Definition** **4** (Replay resistance)**.** *Replay resistance holds if a proof generated for registry version $\eta_{t}$ and data root $R_{\mathrm{data},t}$ is rejected after a valid update to $\left(R_{\mathrm{data},t+1},\eta_{t+1}\right)$, except with negligible probability.*

**Theorem** **3.** *Assuming the registry enforces monotonic nonces and authenticated owner updates, zk-Guard-R rejects stale proofs after data root updates.*

**Proof.**  Every accepted proof has public inputs containing $R_{\mathrm{data}}$ and η. The verifier or gateway obtains the latest tuple from the registry and requires exact equality. After an owner-authorized update, $\eta_{t+1}\neq \eta_{t}$ and, for content changes, $R_{\mathrm{data},t+1}\neq R_{\mathrm{data},t}$ except with a hash collision. A proof bound to the old tuple therefore fails the public-input equality check. A forged update would violate owner-signature authentication or registry monotonicity.    □

**Proposition** **1** (Economic request bound)**.** *Let ϵ be the minimum non-refundable cost charged per accepted chunk release, and let D be the attacker’s locked channel deposit. Ignoring network-level amplification outside the gateway, the attacker can obtain at most ⌊D/ϵ⌋ accepted releases before settlement or channel exhaustion.*

This proposition does not eliminate denial-of-service at the packet layer. It narrows the economically rational attack surface at the application layer: bandwidth-consuming releases are coupled to spendable channel state, and invalid requests can be filtered by deposits, voucher freshness, and proof prechecks.

Malicious gateways are handled by auditability rather than trust. A gateway can deny service, delay release, or equivocate about availability; clients can switch gateways, and signed receipts plus channel state provide dispute evidence for overcharging or replayed vouchers. Collusion between a requester and a gateway cannot forge access because proof verification still uses the current registry tuple, but collusion can reveal the chunks legitimately released to that requester. These cases are now treated as deployment threats rather than hidden by the formal proof.

## 6. Complexity and Experimental Evaluation

### 6.1. Cost Model

Let n=|U| be the attribute universe size, *m* the encoded policy length, $L_{\max}$ the interpreter instruction bound, $S_{\max}$ the stack bound, $d_{A}$ the depth of the attribute commitment tree, $d_{D}$ the depth of the IPFS chunk tree, and *k* the bit width of a range predicate. Let $C_{H}$ denote the constraints for one SNARK-friendly hash, and let $C_{\mathrm{vm}}$ be the per-instruction interpreter cost. For the default Merkle-commitment instantiation, the additional proving constraints over a baseline universal tree circuit are approximately

$$C_{zk-Guard-R}=O\left(d_{A}C_{H}\right)+O\left(d_{D}C_{H}\right)+O\left(L_{\max}C_{\mathrm{vm}}\right)+O\left(kr\right),$$

where *r* is the number of numeric range predicates. The verifier remains constant for Groth16 because all additional checks are inside the proved relation.

For the implemented vector circuit at n=128, the constraint growth is dominated by MiMC-Merkle recomputation. From the measured power-of-two sequence, each additional MiMC hash contributes approximately 826 constraints. The resulting component-level breakdown for the current prototype is shown in Table 3. This table describes the measured vector circuit, not the unimplemented full bytecode interpreter.

### 6.2. Comparison with Matrix-Public zk-Guard

Table 4 summarizes the redesign in a compact capability matrix. The central tradeoff is explicit: zk-Guard-R turns policy privacy, replay resistance, range predicates, and gateway metering into primary mechanisms, while the matrix-public design supports only part of this surface.

### 6.3. Prototype Implementation and Experimental Setup

We implemented a reproducible prototype of the IoT access statement in Go using gnark v0.14.0 and gnark-crypto v0.19.0 over BN254/Groth16. The prototype contains two zk-SNARK circuits. The first circuit models a matrix-public zk-Guard-style baseline in which the policy vector is public and the requester’s attributes are private. The second circuit models the measured zk-Guard-R IoT vector statement: the policy vector is private, MiMC-Merkle policy and attribute roots are public, a numeric age value is committed with MiMC and range checked by bit decomposition, and the proof is bound to a MiMC data Merkle path and nonce. The full reverse-Polish bytecode interpreter remains a design-level component and is not used to support the measured timing claims. MiMC is used because it is a mature SNARK-friendly hash for BN254 in gnark; Poseidon2 support exists in gnark but was not selected for this BN254/Groth16 run because the bundled Poseidon2 test path targets BLS12-377.

All circuit measurements were taken on a Microsoft Windows 11 64-bit local host (build 26200) with an Intel Core Ultra 7 258V processor, 8 cores and 8 logical processors, and 32 GB RAM, using Go 1.23.4 windows/amd64. The original full-size table reports one proof-generation run per attribute size and 20 verification repetitions after circuit compilation and setup; peak memory is the maximum Go heap allocation observed around witness generation and proving. To address runtime variability, we also repeated the n=128 configuration three times with independent compile/setup/prove/verify cycles. For on-chain cost, we exported the gnark BN254/Groth16 Solidity verifier, compiled it with solc 0.8.26, deployed it to a local Foundry Anvil EVM, and recorded transaction receipt gas for verifyProof. The repeated value of 241,942 gas is expected because the deployed verifier has five public inputs independent of *n*; matrix-public verifier gas is not claimed because that verifier was not deployed. Table 5 reports the backend status. We include Circom and Halo2 to make the portability boundary explicit, but only the gnark backend is implemented and measured in this revision.

### 6.4. Experiment 1: zk-SNARK Prototype Performance

**Figure 2:**
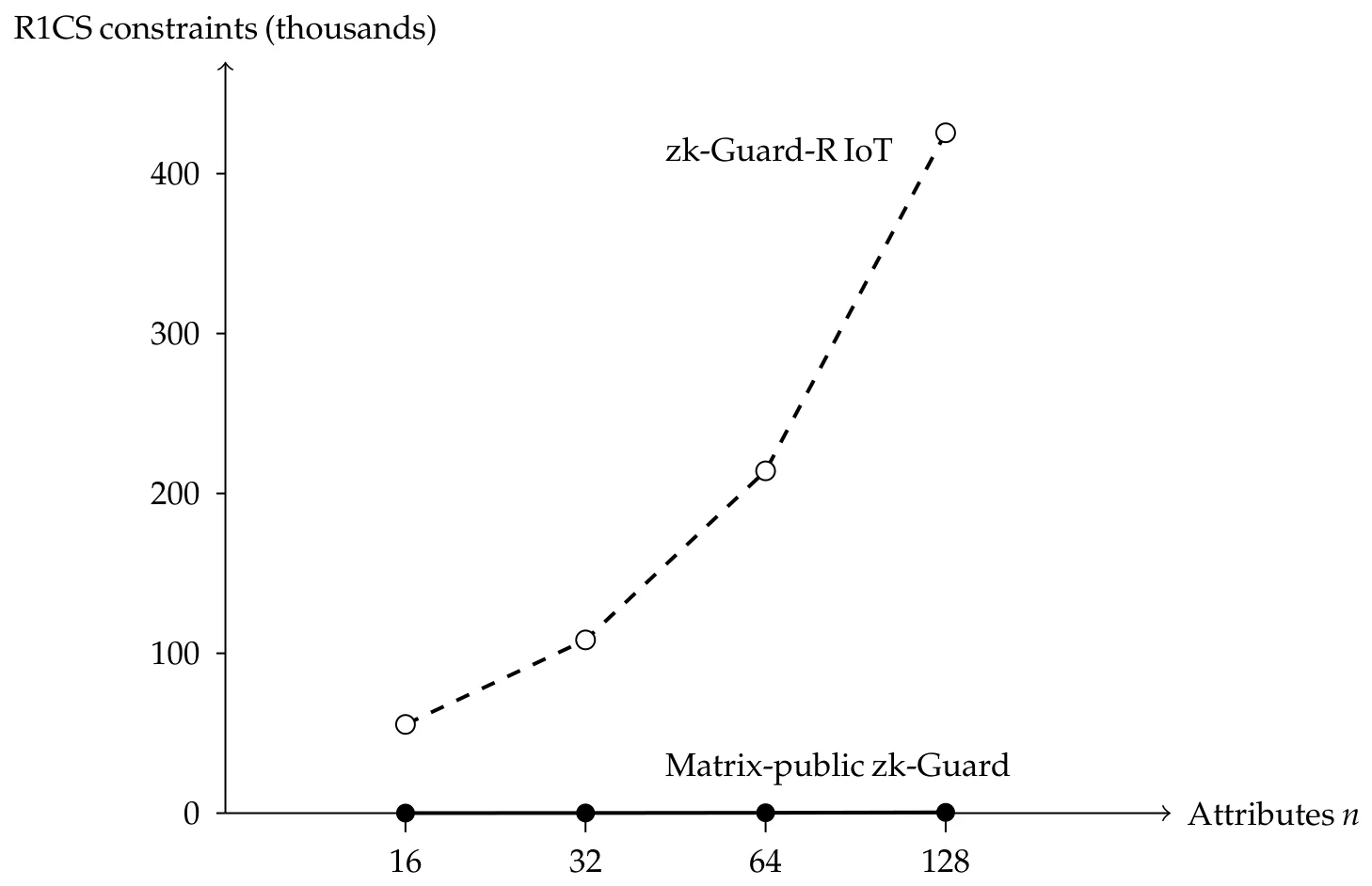
Constraint-count comparison. Real MiMC-Merkle commitments dominate the zk-Guard-R prover circuit, revealing the concrete cost of hiding policy and attribute structure.

**Figure 3:**
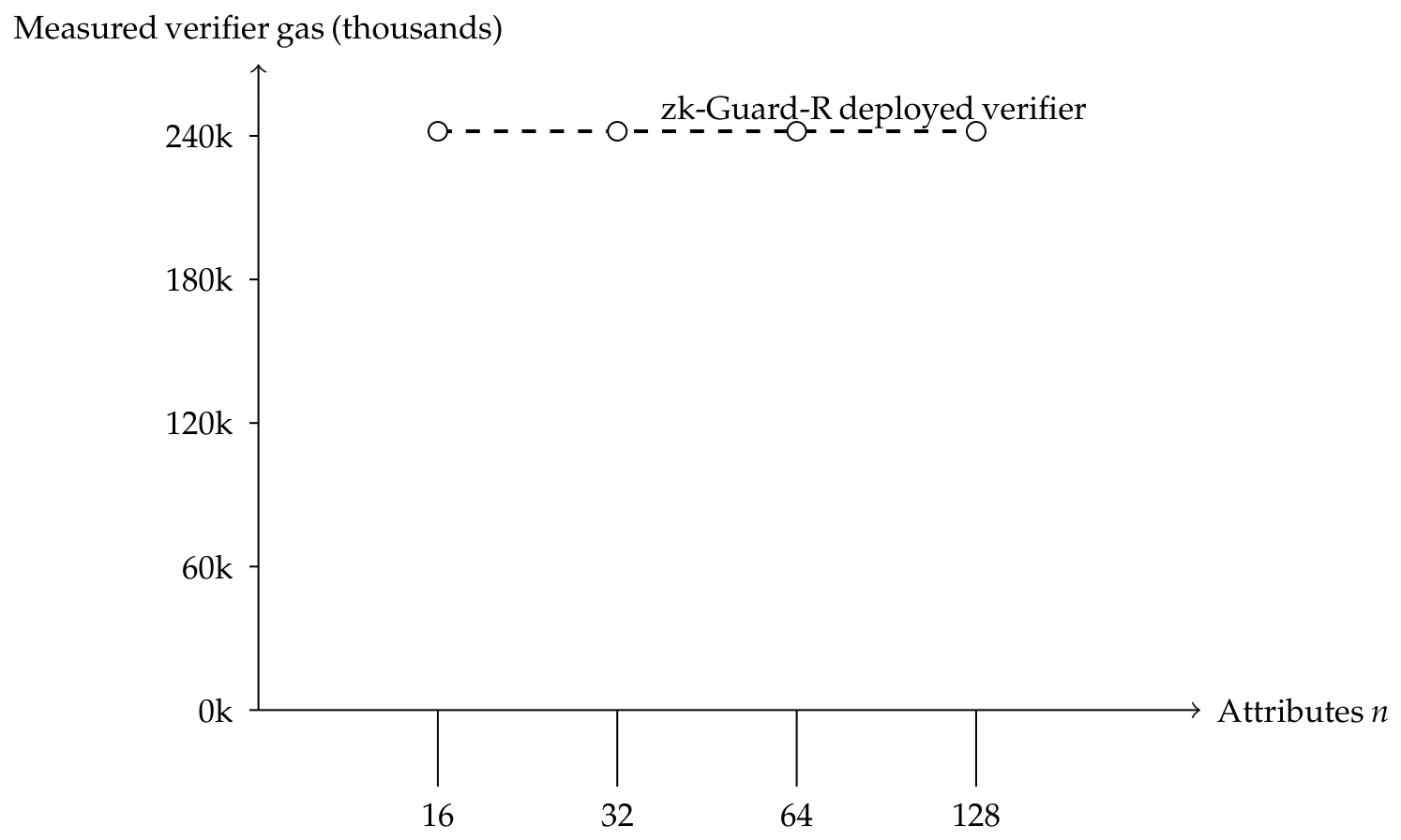
Measured EVM verifier gas for the deployed zk-Guard-R Solidity verifier on a local Anvil EVM. Verification cost remains flat because the public input count is fixed at five.

Table 6 gives the main gnark measurements. With real MiMC-Merkle commitments, the privacy-preserving zk-Guard-R circuit is much larger than the matrix-public baseline, as shown in Figure 2, because every committed policy and attribute leaf is hashed inside the circuit. The important systems effect is that policy structure is hidden and the public-input count stays fixed, as shown in Figure 3. The matrix-public baseline exposes *n* public policy bits, while zk-Guard-R exposes five public values: policy root, attribute root, age commitment, data root, and nonce.

### 6.5. Experiment 2: Baseline Comparison

Table 7 shows the local measurements for the *n* = 128 main configuration. Table 8 compares the 128-attribute zk-Guard-R result with four baselines as a threat-model and cost-context comparison rather than as a claim that all rows are direct substitutes. The blockchain ABAC and IoT token baselines are fast local authorization checks but do not provide hidden policy evaluation or zero-knowledge attribute privacy. The matrix-public zk-Guard prototype is closer in proof system family, but it intentionally exposes the public policy vector and is therefore simpler than the proposed privacy-preserving relation. The CP-ABE row is deliberately labeled as a CP-ABE-style cryptographic-work proxy rather than a complete CP-ABE implementation; it captures linear scalar-field work only and should be replaced by a full CP-ABE library in a deployment study. This disclosure is important because CP-ABE ciphertext/key sizes, pairings, setup, revocation behavior, and update costs depend strongly on the chosen scheme.

### 6.6. Experiment 3: Sensor, Edge, and Mobile-Class Workload Mapping

**Figure 4:**
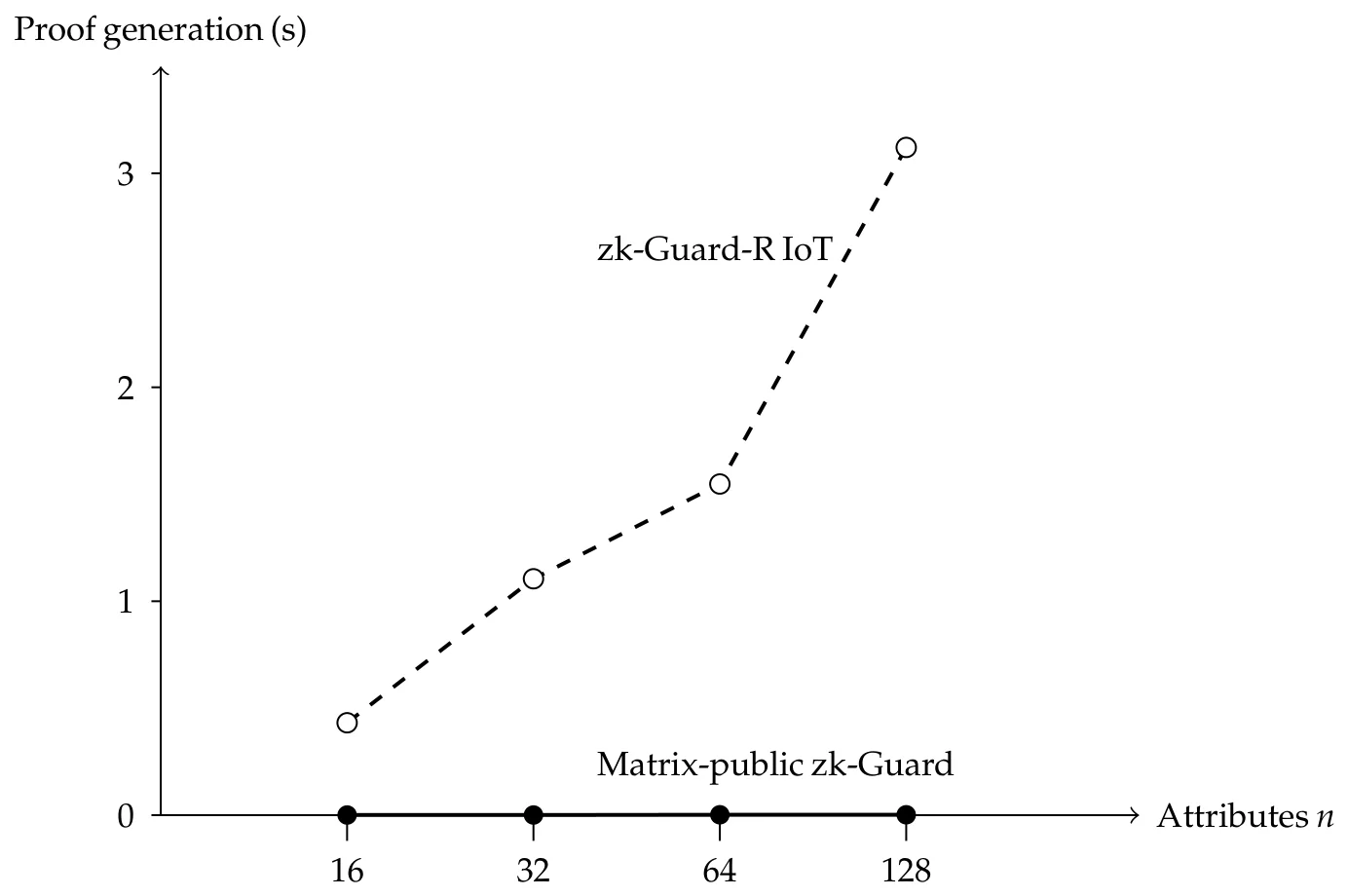
Proof-generation time. Real MiMC-Merkle commitments move the dominant cost to the requester-side prover, while verifier time remains around one millisecond.

The prototype was prepared for physical sensor, edge, and mobile-class experiments by cross-compiling the gnark benchmark for Linux/ARM64 and Android/ARM64 and by adding scripts for SSH-based edge-board runs and ADB-based Android runs. No ARM or Android device was connected during this revision; therefore, we do not report physical-device latency, energy, or memory measurements. Instead, we report the local-host role partition and keep the device scripts and binaries as reproducibility artifacts. A constrained sensor should only encrypt chunks and attach lightweight tokens or signatures; in the prototype, HMAC verification over a 128-byte sensor context took 0.506 microseconds on the local host. The edge gateway performs proof verification and voucher checks; the measured zk-Guard-R verification time was 0.727 ms in the original n=128 run and 0.931±0.127 ms in the three-run repeat. The mobile or edge requester performs proof generation; the original measured proof time was 3121.100 ms at n=128, with 641.491 MB peak Go heap allocation, and the repeated local proof time was 2800.647±539.940 ms with 653.568±22.366 MB peak heap. As shown in Figure 4, these values indicate that the heavy operation is request-side proving, which is the intended partition, but they also show that real ARM/mobile measurements remain necessary before making deployability claims for low-power requesters.

### 6.7. Experiment 4: Replay and Gateway-Abuse Stress Cases

**Figure 5:**
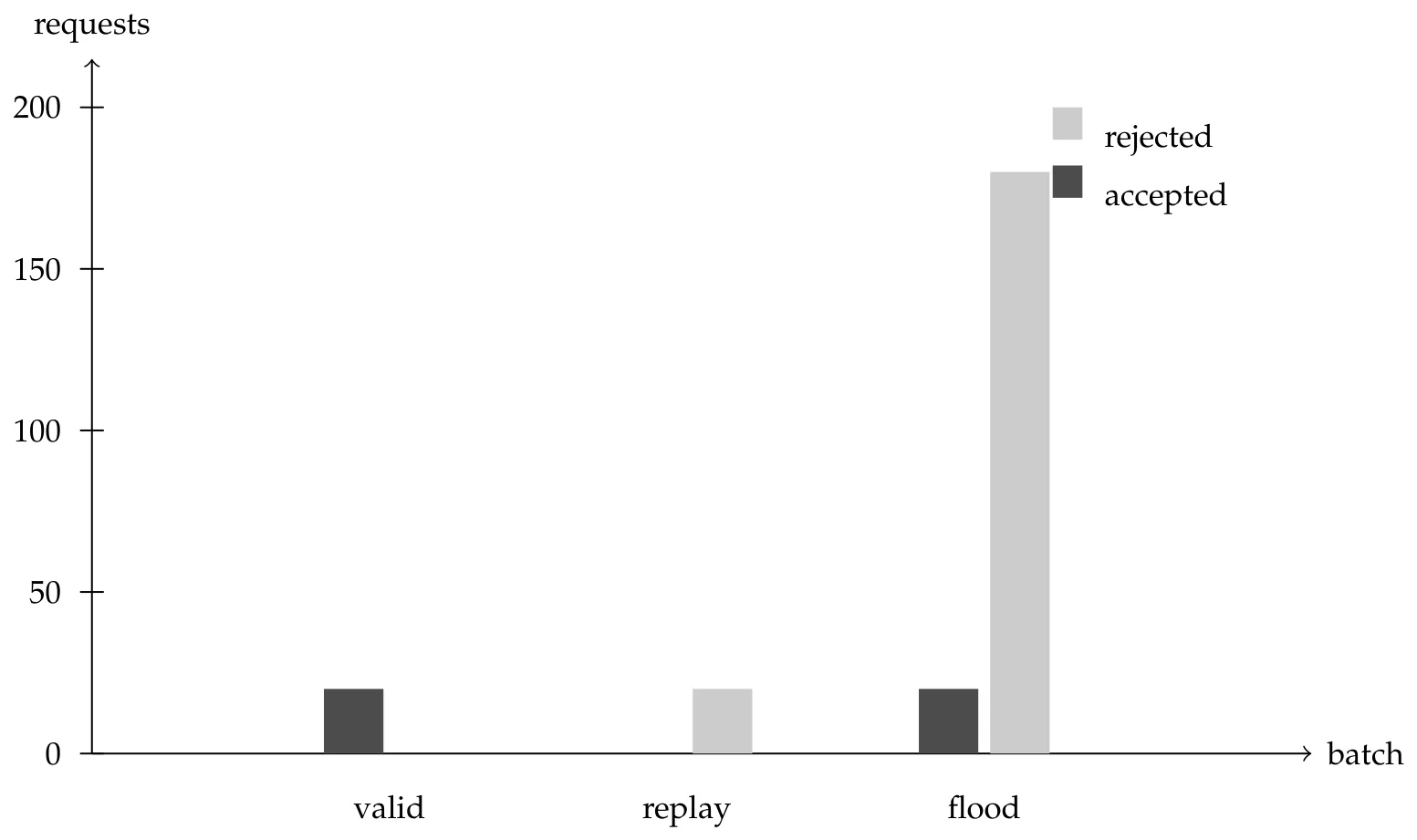
Accepted and rejected requests in the local IPFS gateway stress test. Replay checks reject stale nonces, and voucher accounting bounds accepted flood traffic.

We instantiated the gateway experiment with a real local Kubo/IPFS node and HTTP gateway. A synthetic sensor payload was added to Kubo/IPFS and received CID QmPJsUcQ3X9pmLfubnjR22cCpDQYGMkAqiWCr8rXxHSwct. The direct gateway fetch time for this payload was 83.243 ms. We then placed a local zk-Guard-R gateway proxy in front of the IPFS gateway and tested three request classes, as shown in Figure 5: valid current nonce requests, stale nonce replay requests, and a voucher-limited flood. Table 9 reports the results.

Across all batches, the gateway accepted 40 releases, rejected 20 stale nonce replays, rejected 180 payment-exhausted flood requests, and observed no upstream IPFS errors. These numbers are local single-node stress measurements rather than a public multi-node IPFS deployment, but they validate that freshness and economic metering are executable protocol checks rather than purely analytical assumptions.

## 7. Conclusions

This paper presented zk-Guard-R, a policy-hidden and replay-safe zk-SNARK access control framework for IoT sensor data stored on IPFS. The design replaces public sparse policy matrices with hidden policy commitments, binds access proofs to both logical and physical roots plus a nonce, separates attribute possession from policy interpretation through a bounded stack-based policy VM, supports numeric predicates over committed attributes, and uses payment channel vouchers to meter gateway bandwidth. The resulting architecture keeps succinct public verification while addressing policy side channels, stale MTP replay, policy-update friction, limited predicate expressiveness, and economically free request flooding. The gnark research prototype shows that real MiMC-Merkle policy and attribute commitments substantially increase prover-side cost but keep the public-input count and deployed verifier gas constant as the evaluated attribute universe grows. At n=128, the implemented vector prototype used 425,574 constraints, generated proofs in 3121.100 ms in the original run and 2800.647±539.940 ms over three repeated runs, verified in 0.727 ms in the original run and 0.931±0.127 ms over repeated runs, used approximately 641–654 MB peak Go heap allocation, and consumed 241,942 gas for a deployed Solidity verifier transaction on Anvil. A local Kubo/IPFS gateway stress test further confirmed that stale nonces are rejected before release and that accepted flood traffic is bounded by voucher budget. These values should be read as reproducible prototype evidence. The main remaining engineering tasks are to implement and benchmark the complete bytecode interpreter circuit, benchmark Circom and Halo2 backends, replace the CP-ABE proxy with a complete CP-ABE implementation, export proving and verification key sizes, add concurrent gateway experiments, and repeat the experiments on connected ARM edge and Android/mobile-class devices.

## Figures and Tables

**Table 1:** Core algorithms of zk-Guard-R.

Algorithm	Function
Setup	Input: $1^{\lambda },L_{\max},S_{\max}$. Generates the fixed policy-interpreter circuit, proving key, and verification key for the selected bounds.
RegisterPolicy	Input: P,B. Commits to the private sensor-access policy, computes $R_{\mathrm{logic}}$, builds the encrypted sensor-chunk Merkle root $R_{\mathrm{data}}$, initializes η, and writes the signed registry tuple.
UpdateData	Input: $pid,B^{\prime }$. Re-encrypts or rotates the sensor chunk set as needed, computes $R_{\mathrm{data}}^{\prime }$, increments η, and updates the registry.
ProveAccess	Input: $A,P,b_{i},\rho_{P},\eta$. Generates a zk-SNARK proof that requester attributes satisfy the committed policy, that numeric predicates hold, and that the chunk MTP reconstructs the current sensor data root.
VerifyAccess	Input: $\pi ,x_{\mathrm{pub}}$. Checks the proof and requires $R_{\mathrm{logic}}$, $R_{\mathrm{data}}$, and η to equal the latest registry values.
GatewayRelease	Input: $\pi ,v_{j},mtp_{i}$. Verifies channel freshness, proof validity, and MTP validity before releasing an encrypted IPFS sensor chunk and recording a receipt.

**Table 2:** Concrete instantiation and proof inputs used for the security discussion.

Component	Public Material	Private Witness or Check
Registry state	$pid,C_{P},R_{\mathrm{logic}},R_{\mathrm{data}},\eta ,vk,\sigma_{SO}$	Owner signature authenticates updates; nonce monotonicity is enforced by the registry.
Requester attributes	Attribute-root commitment $H_{A}$ or issuer-authorized root	Issued attributes, requester binding material, and membership or signature witness.
Policy	Padded policy commitment $C_{P}$ and bounds $L_{\max},S_{\max}$	Encoded policy, commitment randomness, and interpreter or vector satisfaction witness.
Sensor chunk	Current $R_{\mathrm{data}}$, chunk identifier request, and Merkle path shape	MTP reconstructs the current data root before release.
Gateway voucher	Channel identifier, sequence number, amount, and gateway receipt	Fresh sequence check, sufficient balance, and settlement/dispute evidence.

**Table 3:** Component-level constraint breakdown for the implemented n=128 zk-Guard-R vector circuit.

Component	Constraints	Interpretation
Policy root	210,630	255 MiMC hashes for the private policy vector.
Attribute root	210,630	255 MiMC hashes for the private attribute vector.
Fixed checks	4314	Data freshness, committed age predicate, nonce binding, and relation glue.
**Total measured circuit**	**425,574**	Matches the main gnark measurements in zk-SNARK Prototype Performance.

**Table 4:** Compact design-level comparison of decentralized access control approaches.

Approach	Privacy Surface			Freshness/Update			Gateway/Verification		
	Attr.	Policy	Range	Data	Nonce	No Setup	MTP	Pay	Const.
Smart-contract ABAC	×	×	∘	∘	∘	•	×	∘	×
CP-ABE access control	∘	∘	×	×	×	∘	×	×	×
Policy-specific zk-ABAC	•	∘	∘	×	×	×	×	×	•
Matrix-public zk-Guard	•	∘	×	∘	×	•	•	×	•
**zk-Guard-R**	•	•	•	•	•	•	•	•	•

•: primary mechanism; ∘: partial or deployment-specific support; ×: not a primary mechanism. “Const.” denotes constant-size public verification with respect to policy size.

**Table 5:** Prototype backend status.

Backend	Prototype Coverage			Reported Evidence				Port.
	Circuit	Prover	Verifier	Constr.	Time	Memory	Gas/IPFS	Trans.
gnark v0.14.0/BN254-Groth16	•	•	•	•	•	•	•	•
Circom/snarkjs/Groth16-compatible	×	×	∘	×	×	×	×	∘
Halo2/PLONKish	×	×	×	×	×	×	×	∘

•: implemented or measured in this revision; ∘: available only through backend translation or ecosystem support; ×: not implemented or not measured. “Port./Trans.” denotes portability through circuit translation or re-arithmetization.

**Table 6:** Measured gnark BN254/Groth16 prototype results.

*n*	Circuit	Constr.	Public	Prove ms	Verify ms	MB	Gas
16	Matrix-public zk-Guard	64	16	1.039	0.520	0.738	–
16	zk-Guard-R IoT	55,518	5	432.373	1.027	95.414	241,942
32	Matrix-public zk-Guard	128	32	1.189	1.034	0.936	–
32	zk-Guard-R IoT	108,382	5	1105.484	1.260	89.450	241,942
64	Matrix-public zk-Guard	256	64	2.013	1.118	99.803	–
64	zk-Guard-R IoT	214,114	5	1547.574	0.997	389.102	241,942
128	Matrix-public zk-Guard	512	128	2.039	1.032	399.455	–
128	zk-Guard-R IoT	425,574	5	3121.100	0.727	641.491	241,942

Gas is the measured Anvil receipt gas for the deployed zk-Guard-R Solidity verifier. Matrix-public gas is not reported because that verifier was not deployed in this run.

**Table 7:** Repeated local measurements for the n=128 main configuration.

Circuit	Runs	Constr.	Public	Setup ms	Prove ms	Verify ms
Matrix-public zk-Guard	3	512	128	86.170 ±34.450	4.443 ± 2.138	0.983 ± 0.431
zk-Guard-R IoT vector prototype	3	425,574	5	28,380.710 ± 3594.365	2800.647 ± 539.940	0.931 ± 0.127

Values are mean ± sample standard deviation over three independent local runs. They are intended to disclose host-side variability, not to replace physical ARM or Android measurements.

**Table 8:** Baseline comparison at n=128.

Scheme	Constr.	Prove ms	Verify/Decision	Verifier Gas	Interpretation
Matrix-public zk-Guard prototype	512	2.039	1.032 ms	–	Real gnark proof, but the policy vector is public and its verifier was not deployed in this run.
**zk-Guard-R IoT prototype**	**425,574**	**3121.100**	**0.727 ms**	**241,942**	Real gnark proof with MiMC-Merkle policy and attribute roots, MiMC data path, age commitment, range check, and nonce binding.
Blockchain ABAC CPU baseline	0	–	0.521 μs	–	Local public-policy decision loop; no policy hiding and no zero-knowledge attribute privacy.
IoT token/HMAC baseline	0	–	0.506 μs	–	Fast token verification for sensor contexts; no expressive hidden policy.
CP-ABE-style crypto proxy	0	–	0.622 μs	–	Linear scalar-field proxy only; not a complete CP-ABE scheme.

Only the zk-Guard-R verifier gas is a deployed-contract measurement; it is the successful verifyProof transaction receipt gas on a local Anvil EVM.

**Table 9:** Local Kubo/IPFS gateway replay and DoS stress results.

Batch	Requests	Accepted	Rejected	Avg. ms	Interpretation
Valid current nonce	20	20	0	4.838	Current $R_{\mathrm{data}}$ and nonce pass the gateway check and fetch the live IPFS object.
Replay with stale nonce	20	0	20	0.208	Stale registry tuples are rejected before chunk release.
Voucher-limited flood	200	20	180	18.453	Accepted releases are bounded by the remaining voucher budget; excess requests are rejected.

## Data Availability

The original contributions presented in this study are included in the article. Further inquiries can be directed to the corresponding author.

## Abbreviations

The following abbreviations are used in this manuscript:

ABE	Attribute-based encryption
CP-ABE	Ciphertext-policy attribute-based encryption
IPFS	InterPlanetary File System
MTP	Merkle tree proof
R1CS	Rank-1 constraint system
SNARK	Succinct non-interactive argument of knowledge
ZKP	Zero-knowledge proof
